# Comparative Evaluation of Bio-C Temp and Calcium Hydroxide as Intracanal Medicaments in Managing Postoperative Pain and Percussion Sensitivity in Symptomatic Irreversible Pulpitis: A Preliminary Prospective Observational Cohort Study

**DOI:** 10.3390/jcm15135274

**Published:** 2026-07-06

**Authors:** Banu Arıcıoğlu, Ahter Şanal Çıkman, Abdullah Özceylan, Hakan Arslan

**Affiliations:** 1 Department of Endodontics, Faculty of Dentistry, Medeniyet University, 34956 Istanbul, Turkey; banu.aricioglu@medeniyet.edu.tr (B.A.); hakan.arslan@medeniyet.edu.tr (H.A.); 2 Department of Endodontics, Faculty of Dentistry, Recep Tayyip Erdogan University, 53100 Rize, Turkey; ahter.sanalcikman@erdogan.edu.tr

**Keywords:** endodontic treatment, bioceramic based medicament, post operative pain, percussion sensitivity

## Abstract

**Objectives**: This prospective observational study aimed to compare the efficacy of a novel bioceramic intracanal medicament (Bio-C Temp) with a conventional calcium hydroxide-based medicament (UltraCal XS) in reducing postoperative spontaneous pain and percussion sensitivity in patients with symptomatic irreversible pulpitis (SIP) with apical periodontitis (AP). **Methods**: Thirty-seven systemically healthy patients diagnosed with SIP with AP were included. The selected teeth were vital, single-rooted, single-canal maxillary or mandibular teeth presenting with prolonged moderate to severe pain in response to cold testing (Visual Analog Scale [VAS] ≥ 50 mm), moderate to severe percussion pain (VAS ≥ 50 mm), and a periapical index (PAI) score ≤ 2. Patients were randomly allocated to receive either Bio-C Temp (*n* = 19) or UltraCal XS (*n* = 18) as the intracanal medicament. Postoperative pain, percussion sensitivity, and analgesic consumption were recorded at 6, 12, and 24 h, and 3, 5, and 7 days. Data were analyzed using R statistical software (version 4.5.0). Statistical significance was set at *p* < 0.05. **Results**: Both groups demonstrated a significant reduction in postoperative pain and percussion sensitivity compared with preoperative levels (*p* < 0.05). By the end of the 7th day, the UltraCal XS group showed significantly lower spontaneous pain (*p* < 0.001) and percussion sensitivity (*p* < 0.001) than the Bio-C Temp group. Complete spontaneous pain relief was reported in 65% of the UltraCal XS group and 31% of the Bio-C Temp. However, no patient in either group achieved complete resolution of percussion sensitivity. **Conclusions**: UltraCal XS provided superior short-term postoperative pain relief and greater reduction in percussion sensitivity compared with the bioseramic medicament. Neither medicament fully resolved percussion sensitivity within 7 days.

## 1. Introduction

The experience of tooth pain is a significant concern for both patients and clinicians before and after root canal treatment. Pain of endodontic origin is often associated with bacterial byproducts, chronic inflammation, activation of the cytokine network, and influx of immune cells [1]. When caries progresses toward the pulp, symptomatic irreversible pulpitis can develop, leading to pulp inflammation and pain triggered by thermal changes or spontaneously. When inflammation extends beyond the root canal system to the periodontal ligament surrounding the root, patients typically experience pain during chewing, percussion, or palpation [2]. This clinical condition is called symptomatic apical periodontitis and may present with or without radiographic evidence of periapical pathology. It is usually characterized by dull or throbbing pain and is often exacerbated by occlusal loading. The affected tooth usually responds negatively or with delayed responses to vitality tests and shows marked tenderness on percussion [3].

Clinical guidelines emphasize the importance of chemomechanical debridement and disinfection of the root canal system for effective infection control in apical periodontitis [4]. Mechanical instruments alone are insufficient to remove all debris from the root canal system; this highlights the critical role of the adjunctive use of intracanal medicaments in addition to irrigation solutions. An ideal intracal medicament should exhibit a broad-spectrum and sustained antimicrobial effect while remaining biocompatible and preventing irritation of periapical tissues. Among the currently available intracanal medications, calcium hydroxide and its combinations have become the most commonly used intracanal medication to relieve inflammation in endodontic practice due to their strong antibacterial properties, ability to neutralize endotoxins, and tissue-dissolving capacity [5]. However, it may not be equally effective against all microorganisms. Furthermore, the literature reports that prolonged use of calcium hydroxide pastes weakens root dentin and that serious tissue reactions may occur in case of extrusion of the material into periradicular tissues [6].

Recently, a bioceramic intracanal medicament (Bio-C Temp, Angelus) was introduced as an alternative to the calcium hydroxide, which is both biocompatible with tissues and can be used as an intracanal medicine thanks to its calcium silicate-based formulation [7]. Bioceramics are advanced agents with promising biocompatibility, anti-inflammatory and sealing properties, with a wide range of clinical applications including regenerative endodontic procedures, root apex fillings and perforation repairs. These medicines not only enhance disinfection but also contribute to improved healing outcomes, making them an integral part of modern endodontic treatment protocols [8]. Particularly in the management of symptomatic teeth, the question has arisen as to whether bioceramic intracanal medicines offer an advantage over calcium hydroxide-based medicines. Despite the clinical significance of postoperative pain, there are no well-controlled clinical studies evaluating the effect of bioceramic-based intracanal medicaments on postoperative pain in symptomatic teeth. The aim of this preliminary prospective observational clinical study was to evaluate the efficacy of a novel bioceramic-based intracanal drug compared to calcium hydroxide in reducing spontaneous pain and percussion sensitivity following root canal debridement in patients with symptomatic irreversible pulpitis and apical periodontitis. The null hypothesis was that there would be no significant difference between the two intracanal drugs in terms of postoperative spontaneous pain and percussion sensitivity.

## 2. Materials and Methods

The study was designed as a cohort study prospectively collecting data from patients diagnosed with symptomatic irreversible pulpitis with apical periodontitis at the Emergency Service of the Department of Endodontics, Faculty of Dentistry of Recep Tayyip Erdoğan University, from June 2025 to July 2025. The study protocol was established by the Ethics Committee of the Faculty of Dentistry at Recep Tayyip Erdoğan University (2025\183; 24 April 2025) and adhered to the STROBE guidelines for observational studies [9].

### 2.1. Participants and Cohort Identification

Systemically healthy patients (ASA I–II) aged 18–60 years diagnosed with symptomatic irreversible pulpitis with apical periodontitis at Emergency Service of the Department of Endodontics, Faculty of Dentistry of Recep Tayyip Erdoğan University were eligible for inclusion in the study. All participants provided written informed consent after receiving detailed information regarding study procedures and potential discomforts. Participation was entirely voluntary, and participants retained the right to withdraw at any time without any repercussions.

The diagnosis of symptomatic irreversible pulpitis and symptomatic apical periodontitis was established according to contemporary endodontic diagnostic criteria described in a standard endodontic textbook [10]. Only vital maxillary or mandibular single-rooted, single-canal teeth exhibiting a prolonged moderate-to-severe pain response (VAS ≥ 50 mm) to cold testing (Hygienic EndoIce, Coltene, Altstätten, Switzerland) were included in the study. Pulp vitality was confirmed using a Digitest II electric pulp tester (Parkell Inc., Edgewood, NY, USA) [10]. The device operates on a scale of 0–64, with 64 representing the maximum stimulus output. The tooth surface was dried, and a conducting medium (toothpaste) was applied before placing the probe on the buccal surface of the tooth. Patients were instructed to indicate the first sensation elicited by the electrical stimulus. The corresponding contralateral healthy tooth, not included in the study, was also tested and served as an internal control for comparison of patient response. The recorded value corresponded to the threshold at which the patient first perceived the stimulus. Each measurement was repeated three times, and the mean value was used for analysis.

Symptomatic apical periodontitis was diagnosed primarily on the basis of clinical findings. Percussion tenderness was assessed by tapping the occlusal surface of the tooth with the back end of a mirror handle, and teeth presenting with moderate to severe percussion pain (VAS ≥ 50 mm) were considered to have symptomatic apical periodontitis. A periapical index (PAI) score ≤ 2 was required to exclude teeth with established radiographic periapical lesions and to ensure inclusion of vital teeth with symptomatic irreversible pulpitis.

Prior to the study, two oral and maxillofacial radiologists were calibrated for PAI scoring using a set of 50 periapical radiographs that were not included in the study sample. The calibration process consisted of independent evaluation of the radiographs according to the original PAI criteria, followed by a consensus session to standardize scoring. During the study, all periapical radiographs were independently assessed by the same calibrated radiologists. In cases of disagreement regarding the PAI score, the final diagnosis was established by consensus involving both radiologists and an experienced endodontist. All dental procedures and clinical diagnostic assessments were performed by a single experienced endodontist.

Patients with systemic disorders requiring antibiotic prophylaxis, psychiatric disorders, allergies to NSAIDs or local anesthetics, use of analgesics or anti-inflammatory drugs within the last 4 h, parafunctional habits such as bruxism, and multiple adjacent teeth requiring endodontic treatment that could cause referred pain were excluded from the study. Teeth with sinus tract or swelling, lack of occlusion with the opposing tooth, or PAI scores greater than 2, periodontal pockets larger than 3 mm, severely damaged teeth, teeth with root resorption or fractures, and teeth that had previously undergone root canal treatment were also excluded.

A total of 106 patients were screened between June and July 2025. Fifty patients diagnosed with conditions other than symptomatic irreversible pulpitis with apical periodontitis, 12 patients with incomplete baseline data, and 5 patients with incomplete informed consent forms were excluded from the study. Consequently, 39 patients were included in the final analysis at the data cut-off date of 1 July 2025.

**Figure 1:**
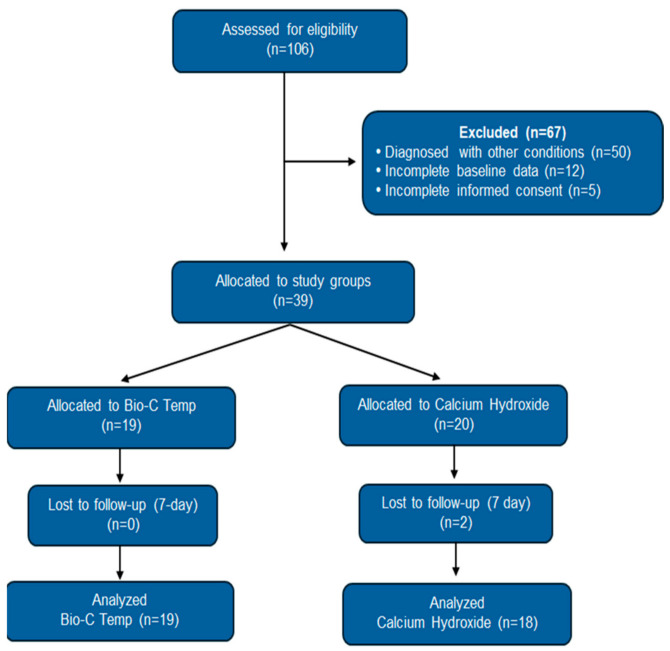
CONSORT-style flow diagram of patient selection, allocation, follow-up, and analysis.

Eligible patients presenting consecutively to the clinic were allocated to receive either a bioseramic-based intracanal medicament (Bio-C Temp; Angelus, Londrina, Brazil) or a conventional calcium hydroxide intracanal medicament (UltraCal XS; Ultradent, South Jordan, UT, USA) using an alternating allocation sequence. Allocation was performed by an independent secretary who was not involved in patient recruitment, treatment procedures, data collection, or outcome assessment. Treatment groups were represented by concealed group codes, and investigators were unaware of treatment assignment at the time of patient enrollment and allocation. Intracanal medicaments were provided to the treating endodontist by a nurse who was not involved in the study, using the concealed group codes. Both the patients and the treating endodontist were unaware of the treatment assignment throughout the study period. The alternating allocation procedure was adopted to maintain balanced group sizes throughout the study period. Because treatment assignment was based on a predetermined alternating sequence rather than random sequence generation, the study was designed as a prospective cohort with alternate treatment allocation. The overall study design and participant flow are presented in Figure 1.

### 2.2. Endodontic Treatment Procedure

Before the treatment, each participant was instructed on how to use the Visual Analog Scale (VAS) [11]. Pain intensity was assessed using a 100 mm horizontal VAS anchored by the verbal descriptors “no pain” (0 mm) and “worst imaginable pain” (100 mm). Patients were asked to place a vertical mark on the line corresponding to their perceived pain intensity. The VAS score was determined by measuring the distance, in millimeters, from the left anchor (0 mm) to the patient’s mark. For descriptive purposes, pain intensity was categorized as follows: no pain (0–4 mm), mild pain (5–44 mm), moderate pain (45–74 mm), and severe pain (75–100 mm).

Percussion tenderness was assessed by the same experienced clinician immediately before treatment and at the end of the 7-day follow-up visit using the VAS. The clinician performing the assessments remained unaware of treatment assignment throughout the study period. Percussion testing was performed by tapping the occlusal surface of the tooth with the back end of a mirror handle. Each assessment was repeated three times at 5 min intervals, and the mean VAS score was used for statistical analysis.

Local anesthesia was administered using 4% articaine hydrochloride with 1:100,000 epinephrine (Ultracaine DS forte; Aventis, Istanbul, Turkey). Aspiration was performed before each injection, and the anesthetic solution was administered only after negative aspiration at a rate of approximately 2 mL/min using a conventional plastic syringe and a 30-gauge, 38 mm needle. A rubber dam was then applied for isolation, and the access cavity was prepared. Canal patency was verified with a #10 K-file (Dentsply Sirona, Ballaigues, Switzerland). The working length was determined using an apex locator (Root ZX II; J. Morita Corp., Tokyo, Japan) and confirmed radiographically.

Root canals were prepared using the ProTaper Next system (Dentsply Maillefer, Ballaigues, Switzerland) according to the manufacturer’s instructions, with the master apical preparation completed three file sizes larger than the initial file reaching the working length. During instrumentation, 2 mL of 2.5% sodium hypochlorite (NaOCl) was used for irrigation after each file, with a size 10 K-file employed to maintain apical patency. The irrigation needle was positioned 2 mm short of the working length to minimize complications.

Final irrigation was performed with 5 mL of 2.5% NaOCl followed by 5 mL of 5% EDTA for 1 min to enhance debridement and smear layer removal. The canals were dried with sterile paper points, and the medicament Bioceramic (Bio-C Temp; Angelus, Londrina, Brazil) or calcium hydroxide (Ultracal XS; Ultradent, South Jordan, UT, USA) was applied using a lentulo spiral (Dentsply Maillefer, Switzerland), placed 1 mm short of the working length. The placement of the medicament was confirmed radiographically. The canal orifice was sealed with sterile teflon tape, and the access cavity was restored temporarily with glass ionomer cement (Kavitan™ Plus; Pentron, SpofaDental, Czech Republic).

Postoperative spontaneous pain levels were assessed using a standardized pain diary provided to each patient. Participants were instructed to record the highest pain intensity experienced at 6, 12, and 24 h and on days 3, 5, and 7 following medicament application. Patients who required analgesics at any time point were instructed to record their VAS score based on the severity of pain immediately before analgesic intake. In case of severe pain, patients were prescribed 400 mg ibuprofen (Brufen; Abbott, Latina, Italy) and instructed to take the medication only if required for pain relief. The timing and number of tablets consumed were recorded throughout the follow-up period. Patients who failed to complete the pain diary were excluded from the study. No other analgesic medications were permitted during the study period.

Patients were scheduled for a recall visit after one week to complete the treatment. The operator performed all clinical outcome assessments, including pain and percussion sensitivity evaluations, before the removal of the intracanal medicament and prior to root canal obturation. Following completion of the assessments, the intracanal medicament was removed and root canal obturation was performed according to the treatment protocol.

### 2.3. Data Collection

All data were collected anonymously by an independent researcher, without reference to patient identifiers. Parameters included patient age and gender, number of emergency department visits, need for nonsteroidal analgesic medication, and pre- and postoperative percussion and spontaneous pain scores.

During the 7-day follow-up period, two participants from the calcium hydroxide group were lost to follow-up due to unavailability for postoperative evaluation. Therefore, 37 patients (19 in the Bio-C Temp group and 18 in the calcium hydroxide group) were included in the final statistical analysis.

### 2.4. Statistical Analysis

Data were analyzed using R statistical software (version 4.5.0). Normality of the distributions was assessed with the Shapiro–Wilk test. Associations between categorical variables were examined using Fisher’s exact test, Fisher’s exact test with Yates’ correction, and the Monte Carlo–corrected Fisher’s exact test. For comparisons of age, which was normally distributed, between two groups, the independent samples *t*-test was applied. Pain scores, which were not normally distributed and varied by group and time, were analyzed using a robust mixed ANOVA implemented in the WRS2 package; post hoc multiple comparisons were conducted using the robust *t*-test with Holm correction. Categorical variables were presented as frequencies and percentages. Continuous variables were reported as mean ± standard deviation, trimmed mean ± standard error, and median (Q1–Q3). Statistical significance was set at *p* < 0.05. The statistical significance level was set at a 95% confidence interval.

## 3. Results

Among the 37 patients included in the study, 16 (43.2%) were female and 21 (56.8%) were male. In the Bio-C Temp group, 52.6% of participants were female and 47.4% were male, whereas in the calcium hydroxide group, 33.3% were female and 66.7% were male. The mean age was 35.95 years in the Bio-C Temp group and 42.28 years in the calcium hydroxide group. No statistically significant differences were observed between the groups with respect to age or gender distribution (*p* > 0.05). The demographic characteristics of the participants are presented in Table 1.

In the Bio-C Temp group, one patient required an unscheduled appointment, whereas no patients in the calcium hydroxide group required an emergency visit. Postoperative analgesic intake was reported by 7 of 19 patients (36.8%) in the Bio-C Temp group and by 5 of 18 patients (27.8%) in the calcium hydroxide group. The distribution of postoperative analgesic intake according to follow-up time point was given in Table 2. No statistically significant differences were observed between the groups regarding overall analgesic intake (*p* > 0.05) or analgesic intake at any postoperative time point (*p* > 0.05).

No statistically significant difference was observed between the groups in terms of baseline percussion sensitivity (*p* > 0.05). During the postoperative follow-up period, percussion sensitivity decreased significantly in both groups compared with baseline values (*p* < 0.001). At the 7-day evaluation, lower percussion sensitivity scores were observed in the calcium hydroxide group than in the Bio-C Temp group (*p* = 0.041). However, no patient in either group achieved complete (100%) resolution of percussion sensitivity by postoperative day 7 (Table 3).

Regarding spontaneous pain, there was no statistically significant difference between the groups in preoperative pain scores, as indicated by the same uppercase letter in the preoperative row. However, the robust mixed ANOVA revealed a significant overall group effect (*p* = 0.004), a significant time effect (*p* < 0.001), and a significant group × time interaction (*p* = 0.001), indicating that spontaneous pain changed over time differently between the groups. Pain scores decreased significantly and consistently over time compared to baseline values. By the end of the 7-day evaluation, the calcium hydroxide group demonstrated significantly lower pain scores than the Bio-C Temp group (*p* < 0.001), and the greatest difference was observed on days 3 and 5 postoperatively (*p* < 0.001). Complete pain relief (VAS = 0) was observed in 65% of patients in the calcium hydroxide group, while this rate was 31% in the Bio-C Temp group.

Intragroup analysis revealed that the Bio-C Temp group exhibited statistically significant differences between postoperative 12 h and day 1, and also between days 5 and 7. Meanwhile, the calcium hydroxide group showed significant changes between 6 and 12 h and 12 h and day 1, as well as across the intervals of days 1–3, 3–5, and 5–7 (*p* < 0.001) (Table 4).

## 4. Discussion

Endodontic pain is a clinical manifestation of the local inflammatory response of the periapical tissues [12]. This response involves a complex cascade of biochemical events triggered by mechanical, chemical, or microbial irritation during root canal procedures. The standard protocol for endodontic disinfection involves the complete removal of infected debris from the root canal system by mechanical instruments and abundant irrigation, followed by the placement of an intracanal medication [13]. Calcium hydroxide is widely regarded as a universal intracanal medication used for this purpose [14]. Its antimicrobial effect results from its ionic dissociation into calcium and hydroxyl ions in aqueous solution [5], and it has also been suggested to exert analgesic effects by modulating inflammation and promoting tissue repair [15]. Despite its biological advantages, it may not be equally effective against all microorganisms present in the root canal system, and its disadvantage is that its long-term use weakens the root dentin and causes serious tissue reactions if extruded into the periradicular tissues [16]. Bioceramic intracanal medicaments, particularly based on calcium silicates, have gained increasing attention due to their high biocompatibility and bioactivity [17,18]. It is a ready-to-use paste containing calcium silicate, calcium tungstate, titanium oxide, and other compounds, and is presented in a resin-based carrier that prevents hydration and hardening [19]. Unlike traditional calcium hydroxide formulations, it does not require frequent reapplication. The manufacturer claimed that Bio-C Temp can be used as an intracanal medicament as an alternative to calcium hydroxide pastes [18].

Although several in vitro studies have been conducted on Bio-C Temp, there is limited clinical evidence comparing its effectiveness to other intracanal medications. Therefore, this prospective observational study aimed to compare the effects of traditional calcium hydroxide and bioceramics-based intracanal medications on spontaneous pain and percussion sensitivity in teeth with symptomatic irreversible pulpitis and apical periodontitis after root canal cleaning.

In the present prospective cohort study with alternate treatment allocation, both calcium hydroxide and bioceramic intracanal medicaments were associated with a reduction in spontaneous pain and percussion sensitivity over time. At the end of the first week, lower spontaneous pain and percussion sensitivity scores were observed in the calcium hydroxide group compared with the bioceramic group. Although these differences reached statistical significance, the absolute magnitude of the between-group differences was relatively small. From a clinical perspective, both intracanal medicaments were associated with substantial symptom reduction over the follow-up period.

The biological activity of intracanal medicaments is closely related to their ability to generate an alkaline environment [20]. The calcium hydroxide paste used in this study, UltraCal XS (Ultradent Products, South Jordan, UT, USA), contains 35% calcium hydroxide powder in an aqueous vehicle, providing the rapid dissociation and release of calcium and hydroxyl ions, resulting in a high pH of approximately 12–12.5 [21]. In contrast, the Bio-C Temp bioceramic material does not contain calcium hydroxide as a direct component; instead, it forms calcium silicate gel and calcium hydroxide upon hydration, leading to the gradual release of Ca^2+^ and OH^−^ ions. The pH of the medium gradually increases, reaching approximately 10.79 after seven days [22]. This difference in ion release kinetics may partly explain the more rapid reduction in symptoms observed in the calcium hydroxide group.

In contrast to the current findings, a previous study reported no significant differences between calcium hydroxide and bioceramic intracanal medicaments with respect to postoperative pain at different follow-up time points [23]. These discrepancies may be explained by differences in study design and case selection. The previous study was conducted on previously root canal treated teeth with apical periodontitis and relatively mild pain levels, whereas the present study involved vital teeth with symptomatic apical periodontitis presenting with moderate to severe pain. These differences in baseline clinical characteristics may have influenced the observed outcomes.

Another important finding of the study was the progressive reduction in pain intensity was observed in both groups, particularly on days 3 and 5, which is consistent with previously published literature [24]. Since the mechanisms of post-treatment pain in different parts of the body are similar, it can be attributed to a similar mechanism. Pain usually peaks within the first 24–48 h because this coincides with the acute inflammatory phase following tissue irritation. After instrumentation or drug administration, tissues release chemicals such as prostaglandins, bradykinin, histamine, and cytokines (such as IL-1, TNF-α) [25]. Simultaneously, inflammation causes vasodilation, fluid accumulation, and increased tissue pressure in the limited periodontal ligament space, making the tooth particularly sensitive to biting or pressure. This inflammatory response usually reaches its peak intensity within the first two days and then begins to decrease; Therefore, many studies [15,26,27,28,29,30] report that pain peaks within 24–48 h and then gradually decreases after approximately 72 h as inflammatory mediators decrease, tissue pressure decreases, healing begins, and the antimicrobial effects of the drugs become apparent [31].

In this study, at the end of 7 days, 31% of participants in the Bio-C Temp group and 65% of participants in the calcium hydroxide group reported complete spontaneous pain relief. Although both medicaments were associated with reductions in percussion sensitivity after 7 days, complete resolution was not observed in any patient. This difference between spontaneous pain relief and percussion sensitivity may be explained by the distinct biological origins of these symptoms. Spontaneous pain is typically associated with pulp inflammation, as observed in cases of symptomatic irreversible pulpitis. This symptom results from increased intrapulpal pressure and subsequent activation of nociceptors within the pulp tissue [32]. In contrast, percussion pain originates primarily from the periapical tissues rather than the pulp itself. As pulpal inflammation extends beyond the apical foramen, it may trigger a periapical inflammatory response, leading to mechanical sensitivity. Consequently, percussion sensitivity may persist even after removal of the inflamed pulp because of ongoing inflammation in the periapical tissues [33]. Management of persistent inflammation requires removal of infected pulp tissue and the use of intracanal medicaments with sustained antimicrobial activity, partly mediated by a localized increase in pH within the root canal system [34]. Previously, it has been suggested that calcium hydroxide should remain within the root canal for approximately 7 days to achieve its full antimicrobial effect [35]. Accordingly, a 7-day follow-up was selected in the present study, as this interval is commonly used in clinical investigations evaluating the short-term effects of intracanal medicaments. However, it has been reported that although pH in the inner dentin increases rapidly within hours, peak pH values in the peripheral dentin are not reached until approximately 2–3 weeks after calcium hydroxide placement weeks [36]. Additionally, it was indicated that the buffering capacity of dentin, the time required for hydroxyl ion diffusion into dentinal tubules, and anatomical complexities of the root canal system may have further limited the antimicrobial action of the medicament [37]. Therefore, the 7-day follow-up period used in the present study may not have been sufficient to capture the full biological effect of the medicament, which may partly explain why percussion sensitivity was not completely resolved in either group. Prolonging the contact time between dentin and the medicament may have facilitated deeper penetration into dentinal tubules and enhanced treatment efficacy.

Percussion testing presents inherent limitations. A major drawback is the variability in the vertical or lateral forces applied by different clinicians, which introduces heterogeneity in the stimuli. In this study, although all percussion assessments were performed by the same blinded clinician and repeated three times at 5 min intervals to improve measurement consistency, the applied percussion force was not instrumentally standardized; therefore, some degree of measurement variability cannot be excluded. Moreover, differences in bite forces related to age, sex, craniofacial deformities, and temporomandibular joint disorders further complicate interpretation and compromise the objectivity of these tests [38]. Although the specificity and sensitivity of these methods remain uncertain, several studies suggest that they are readily applicable and can provide confirmatory clinical information [39,40].

In addition, the relatively small sample size and the absence of an a priori sample size calculation may have limited the statistical power of the study to detect differences in certain outcomes, particularly secondary outcomes such as analgesic intake. Analgesic intake may have influenced patient-reported postoperative pain scores. However, patients who required analgesics were instructed to record their VAS scores based on the severity of pain immediately before taking the medication. This approach was intended to minimize the potential influence of analgesics on pain reporting at the specified time point. Furthermore, no significant difference was observed between the groups regarding analgesic consumption, and analgesic intake occurred within the first 6–24 h after treatment. Therefore, it is unlikely that analgesic use substantially influenced the VAS scores recorded during the follow-up period.

Another consideration is that the two participants in the calcium hydroxide group were lost to follow-up and were excluded from the final analysis because outcome data were unavailable. Although the number of missing cases was small, differential loss to follow-up between groups may have introduced bias. Therefore, the findings should be considered preliminary and interpreted with caution.

From a clinical perspective, both intracanal medicaments were associated with reductions in postoperative pain and percussion sensitivity. The greater reduction in symptoms observed in the calcium hydroxide group suggests that intracanal medicament selection may influence short-term symptom management in patients with symptomatic irreversible pulpitis and apical periodontitis. However, given the limited sample size and non-randomized study design, these findings should be interpreted with caution and should not be considered definitive. Future multicenter randomized controlled trials with larger sample sizes, longer follow-up periods, and standardized objective assessment methods are required to validate and extend these findings.

## 5. Conclusions

Within the limitations of this prospective non-randomized cohort study, both intracanal medicaments were associated with substantial reductions in spontaneous pain and percussion sensitivity in teeth diagnosed with symptomatic irreversible pulpitis and apical periodontitis. Calcium hydroxide was associated with greater reductions in spontaneous pain and percussion sensitivity at the 7-day follow-up; however, the observed between-group differences were relatively modest. Given the observational design, alternate allocation method, and limited sample size, these findings should be interpreted with caution. Both medicaments may represent reasonable options for intracanal medication.

## Figures and Tables

**Table 1:** Demographic characteristics of the study participants.

Variable	Bio-C Temp (*n* = 22)	UltraCal XS (*n* = 23)	*p*-Value	ES [95% CI]
Age (years), mean ± SD	35.95 ± 9.76	42.28 ± 12.46	0.093 *	−0.568 [−1.222: 0.095]
Gender (Female), *n* (%)	12 (52.6%)	12 (33.3%)	0.394 **	0.195 [0.010: 0.491]
Gender (Male), *n* (%)	10 (47.4%)	11 (66.7%)		

* Independent Samples *t*-Test; ES [95% CI] = Effect Size [95% Confidence Interval]: Cohen’s d (d). ** Yates’s correction; ES = Effect size; 95% confidence interval was calculated using the bootstrap method; Values are presented as *n* (%).

**Table 2:** Distribution of postoperative analgesic intake according to follow-up time point.

Analgesic Intake	Bio-C Temp	Ultracal XS	Total	*p*	ES [95% CI]
Postop 6th hour					
Presence	3 (15.8)	2 (11.1)	5 (13.5)	1.000*	0.068 [0.004: 0.241]
Absence	16 (84.2)	16 (88.9)	32 (86.5)		
Postop 12th hour					
Presence	2 (10.5)	2 (11.1)	4 (10.8)	1.000 *	0.009 [0.004: 0.009]
Absence	17 (89.5)	16 (88.9)	33 (89.2)		
Postop 24th hour					
Presence	2 (10.5)	1 (5.6)	3 (8.1)	1.000 *	0.091 [0.004: 0.247]
Absence	17 (89.5)	17 (94.4)	34 (91.9)		

* Fisher’s exact test; ES = effect size; 95% confidence interval was calculated using the bootstrap method; *n* (%).

**Table 3:** The distribution of percussion sensitivity scores.

	Group		Total		Test Statistic	*p*	ES (95% CI)
	Bio-C Temp	UltraCal XS					
Preoperative percussion	57.89 ± 1.49 ^a,A^	57.50 ± 1.81 ^a,A^	57.71 ± 1.11	Group	45.258	0.041	0.116 (0: 0.320)
Postoperative Day 7	13.95 ± 2.39 ^b,A^	5.00 ± 1.35 ^b,B^	9.29 ± 1.60	Time	51,607.268	<0.001	0.993 (0.988: 0.995)
Total	35.97 ± 4.25	31.03 ± 5.05	33.53 ± 3.28	Group × Time	405.816	<0.001	0.543 (0.293: 0.682)

Robust mixed ANOVA; trimmed mean ± standard error (5% trimming ratio); a and b: No difference between times with the same letter within each group; A and B: No difference between groups with the same letter within each row. Effect Size (95% CI): Partial eta squared (95 %confidence interval).

**Table 4:** Intergroup comparison of spontaneous pain scores over time.

	Group		Total		Test Statistic	*p*	ES (95% CI)
	Bio-C Temp	UltraCal XS					
Preoperative	65.00 ± 1.47 ^a,A^	65.56 ± 2.15 ^a,A^	65.14 ± 1.27 ^a^	Group	94.412	0.004	0.212 (0.025: 0.416)
Postoperative 6th hour	53.95 ± 1.93 ^b,A^	51.11 ± 2.44 ^b,A^	52.71 ± 1.39 ^b^	Time	5020.105	<0.001	0.991 (0.980: 0.993)
Postoperative 12th hour	50.26 ± 2.64 ^b,A^	42.78 ± 2.43 ^b,A^	47.00 ± 1.79 ^c^	Group × Time	54.355	0.001	0.555 (0.154: 0.649)
Postoperative Day 1	35.53 ± 2.91 ^c,A^	28.89 ± 1.71 ^c,A^	32.29 ± 1.55 ^d^				
Postoperative Day 3	28.16 ± 3.21 ^cd,A^	15.83 ± 1.92 ^d,B^	21.29 ± 1.72 ^e^				
Postoperative Day 5	18.42 ± 1.70 ^d,A^	8.33 ± 1.19 ^e,B^	13.43 ± 1.39 ^f^				
Postoperative Day 7	6.68 ± 1.83 ^e,A^	1.94 ± 0.91 ^f,A^	4.06 ± 1.12 ^g^				
Total	36.96 ± 2.04	29.91 ± 2.23	33.54 ± 1.52				

Robust mixed ANOVA; trimmed mean ± standard error (5% trimming ratio); a–g: No difference between times with the same letter; A and B: No difference between groups with the same letter within each row; Effect Size (95% CI): Partial eta squared (95% confidence interval).

## Data Availability

The original contributions presented in this study are included in the article. Further inquiries can be directed to the corresponding author.
